# Corundum Files, Slabs, and Wheels

**Published:** 1853-10

**Authors:** 


*Corundum Files, Slabs, and Wheels.*—We have received from S. Wardle & Co., Cincinnati, Ohio, some very superior corundum files, slabs, and wheels, of their own manufacture. They are incomparably better than instruments of the same kind made of emery, and we find them fully equal to those obtained from England. Every dentist, having any thing to do with artificial teeth, will find it greatly to his advantage to use them. We believe that S. Wardle & Co. are the only persons, except Jones, White & McCurdy, who manufacture these articles in the United States, and for the very handsome assortment which they were so kind as to send us, we beg to return our thanks.

